# Conditional Mediation of Absorptive Capacity and Environment in International Entrepreneurial Orientation of Family Businesses

**DOI:** 10.3389/fpsyg.2018.00102

**Published:** 2018-02-08

**Authors:** Felipe Hernández-Perlines, Wenkai Xu

**Affiliations:** Department of Business Administration, Faculty of Legal and Social Science, University of Castilla-La Mancha, Toledo, Spain

**Keywords:** international entrepreneurial orientation, absorptive capacity, environment, international performance, conditional mediation, family businesses

## Abstract

This study analyzes the effect of conditional mediation of environment-absorptive capacity in international entrepreneurial orientation of family businesses. Results involve data from 218 Spanish family businesses, analyzed with SmartPLS 3.2.7 software. This paper presents a relevant contribution both to the academic field and the performance of family firms, helping to understand the process of transforming international entrepreneurial orientation into a better international performance through absorptive capacity while family businesses invest their efforts in aligning international entrepreneurial orientation and absorptive capacity with international results, bearing in mind the positive moderator effect of environment. The most relevant contribution of this work is to integrate in the same model the mediating effect of the absorption capacity and the moderating effect of the environment: the effect of the international entrepreneurial orientation on the international performance of family businesses improves with the mediation of the absorptive capacity (the variability of international performance goes from 32.5 to 40.6%) and the moderation of the environment (to variability of international performance goes from 40.6 to 45.3%).

## Introduction

In the past few years, entrepreneurial orientation, internationalization (IEO), absorptive capacity (ACAP), and environment (ENV) have drawn much academic and business interest. This study attempts to address the above concerns by checking how these concepts relate to each other. The first two concepts can be related through what has come to be known as “international entrepreneurial orientation.” It is an emerging area of research that seeks to analyze the innovative, risky and proactive behaviors of companies with international activity (Kropp et al., 2006). There are two ways of to contextualize IEO (Covin and Miller, 2014): (a) it can put into operation by using traditional scales in an international context, or (b) it can be conceptualized as a subcategory of entrepreneurship. This study has opted for the former option, that is, regarding IEO as a similar construct to entrepreneurial orientation, only framed in an international context. In order to make this construct operative, the study used the scale proposed by Miller (1983), Covin and Slevin (1989), and Covin and Miller (2014). With everything, we pose the following research question: Does the international entrepreneurial orientation influence the international performance of family businesses?

This study responds to the research request made by Rauch et al. (2009), Wales et al. (2011), and Covin and Miller (2014), to further analyze entrepreneurial orientation considering other variables that may mediate or moderate. First, we analyze the mediating effect of ACAP. ACAP has been selected due to its relevance for firms when competing in an increasingly competitive environment (Jansen et al., 2005). This effect has already been analyzed in a previous work by Hernández-Perlines et al. (2017) for this group of family businesses. The difference with respect to said work is the dependent variable: in the work of Hernández-Perlines et al. (2017), the dependent variable was the performance of family businesses, in this research the dependent variable is the international performance of family businesses. In short, the second research question: does ACAP mediate the influence of IEO on the international performance of family firms?

Both IEO and ACAP have been analyzed at a company level (Covin and Lumpkin, 2011).

The fourth element that has been considered in this study is the ENV. It plays a remarkable role in the development of IEO (Covin and Slevin, 1991; Zahra et al., 1997; Kreiser et al., 2010). Among published papers, there are some outlining studies analyzing the moderator effect of ENV on entrepreneurial orientation of companies which put into practice strategies of internationalization (Miller, 1983; Russell and Russell, 1992; Tan, 1996; Balabanis and Katsikea, 2004). To analyze the moderating effect, we pose the third research question of this work: does the ENV moderate the mediating effect of ACAP on the influence of IEO on the international performance of family businesses?

The firms under study are family businesses located in Spain. The reason behind this choice is that family businesses, as happens in other countries, represent a major part of the productive system. Spanish family businesses represent 88.8% of all active companies, 57.1% of GVA and 66.7% of private employment (Corona and Del Sol, 2016). Thus, this type of business is an important engine of growth and welfare (Astrachan and Shanker, 2003; Sirmon et al., 2008).

To analyze results and contrast hypotheses, this study suggests a PLS-SEM model of structural equations, using SmartPLS 3.2.7 software (Ringle et al., 2015). Data is collected from the answers to a questionnaire sent by email to the CEOs of family businesses registered in the Spanish Institute for Family Business. Data collection took place during the months of June to November 2016 and valid information was obtained from 218 Spanish family businesses.

This study is structured as follows: after the introduction, the most important literature on IEO, ACAP and ENV will be reviewed, and the hypotheses that have been considered in the research model designed will be considered. In the materials and methods section, the sample is described, as have been measured the different variables considered and the method of analysis used to test the hypotheses. In the results section, the hypotheses are tested and the results obtained in the conditional mediation model proposed are analyzed. Finally, in the discussion section, the main conclusions of the research work are offered and the main limitations of the work are highlighted and future lines of research are indicated.

## Theoretical background and research hypotheses

### International entrepreneurial orientation

Although research in the field of business internationalization has made considerable progress in recent years, the challenge of responding to questions that arise as a result of an increasingly global and competitive business environment still persists (Werner, 2002).

Previous studies have proven the existence of a positive relationship between entrepreneurial orientation and performance (Miller, 1983; Covin and Slevin, 1989; Barringer and Bluedon, 1990; Zahra, 1991; Zahra and Covin, 1995; Wiklund, 1999; Wiklund and Shepherd, 2005), so entrepreneurial orientation is considered a valuable predictor of business success (Kraus et al., 2012).

In business organization literature, the study of internationalization has been addressed from different approaches^1^; although, in recent years, the entrepreneurship approach has emerged strongly. This approach has a high explanatory power of the process of creating value by companies operating abroad (Jones and Coviello, 2005; Weerawardena et al., 2007; Joardar and Wu, 2011). This is how the concept of IEO arises, as a different, dynamic way of explaining why companies become internationalized (e.g., Freeman and Cavusgil, 2007; Sundqvist et al., 2012).

Most studies analyze the influence of IEO in the overall performance of the company. However, many companies, in order to maintain and even improve their competitiveness, seek to develop their business beyond national borders (Autio et al., 2000; Sapienza et al., 2006), thus reducing their dependence on domestic or national markets (Ciravegna et al., 2014). Therefore, one of the most important contributions of this study is to analyze the influence of IEO in international business performance. The latter is measured based on a multi-item scale, which includes international intensity, perceived satisfaction in the international activity and internationalization results (Balabanis and Katsikea, 2004; Dimitratos et al., 2004; Etchebarne et al., 2010). The approach used aims at shedding light on the explanatory power of entrepreneurial orientation from a different, dynamic perspective when analyzing the process of internationalization (Hernández-Perlines et al., 2016a). Based on this, the first research hypothesis can be drawn:

**H_1_: IEO positively influences the international performance of family businesses**.

### Absorptive capacity

What the different authors analyze is not so much how IEO influences business profits, but rather which factors affect this relationship. Lumpkin and Dess (1996) had already analyzed the effect of a number of factors on entrepreneurial orientation in relation to business performance. Some other research studies have explored the role of internal factors such as availability of resources (Wiklund and Shepherd, 2005), marketability, strategy formation process (Covin et al., 2006; García-Villaverde et al., 2013), internal social context (De Clercq et al., 2010), family generations involved in management (Chirico et al., 2011), the effect of technology (Knight, 2000), business capabilities (Ahimbisibwe and Abaho, 2013), the competitive strategy (Hernández-Perlines et al., 2016a) and a set of internal factors (Balabanis and Katsikea, 2004).

Other research studies have shown the role of external factors such as hostility, turbulence and dynamic environment^2^ (Covin and Covin, 1990; Namen and Slevin, 1993; Dess et al., 1997; Wiklund and Shepherd, 2005), the life cycle of industry (Lumpkin and Dess, 2001), external networks (Lee et al., 2001; Stam and Elfring, 2008) or the effect of industry and the market (Lohrke et al., 2015). Finally, Dess et al. (1997) integrated internal and external factors into a configurational model.

This study focuses on the mediating effect of ACAP in the influence of entrepreneurial orientation on business performance. ACAP has been selected due to its relevance for firms, which -in order to survive certain pressures- are forced to recognize, assimilate and apply new knowledge (Jansen et al., 2005). ACAP arises as a key research topic in business strategy (Jansen et al., 2005). The concept of ACAP was originally developed by Cohen and Levinthal (1990). These authors define it as the ability of firms to identify, assimilate and exploit new knowledge. It is an essential intangible asset for success and depends on the level of prior knowledge, which will facilitate the identification and processing of new knowledge. However, ACAP has undergone several reformulations. Zahra and George (2002) revived the interest in this concept by reviewing a number of research studies on the topic and offering a reconceptualization, as a result of integrating previous findings. To them, ACAP is “a set of organizational routines and processes by which firms systematically acquire, assimilate, transform and utilize knowledge” (Zahra and George, 2002, p. 186).

Zahra and George's reconceptualization (2002) has led to plenty of literature on ACAP (Volberda et al., 2010). There are studies addressing the multidimensional nature of ACAP (Jansen et al., 2005; Lane et al., 2006; Todorova and Durisin, 2007) and others analyze the background of ACAP (Kogut and Zander, 1992; Lyles and Salk, 1996; Lane and Lubatkin, 1998; Dijksterhuis et al., 1999; Van den Bosch et al., 1999; Argote and Ingram, 2000; Lane et al., 2001; Lenox and King, 2004; Andersen and Foss, 2005).

The mediating effect of ACAP has been extensively analyzed in the literature. Among others, we highlight the work of Van den Bosch et al. (1999), who analyzed the mediating effect of the ACAP between new knowledge and adaptation of the organization. On the other hand, Wu et al. (2010) studied it in technology management capacity and new product development performance. Adisa and Rose (2013) analyze this mediating effect in the transfer of knowledge; Liu et al. (2013) in IT capabilities on the performance of the company; Wang and Chen (2013) in human resources practices and organizational innovation performance; Saenz et al. (2014) in innovative capacity and buyer-supplier relationships and, Aljanabi et al. (2014) analyzed the mediating effect of the ACAP between organizational support factors and technological innovation. Finally, Leal-Rodríguez et al. (2014) examined the ACAP in relation to the results of innovative capacity. The ACAP also acts as a mediator between the innovation and the performance of the firm (Ferreras-Méndez et al., 2015), or between the training and the performance of the firm (Hernández-Perlines et al., 2016b). The ACAP manages to improve performance when used in combination with innovation capacity (Tzokas et al., 2015). In this sense, the work of Yá-ez-Araque et al. (2017), proposes a model of double mediation of the ACAP and the innovative capacity between training and performance.

The EO has a positive effect on the performance of the company when combined with high levels of ACAP, potential and realized (Sciascia et al., 2014). In an earlier paper by Hernández-Perlines et al. (2017), the mediation of ACAP in the influence of IEO on the performance of family firm is already analyzed. In the present work, the same database is shared: 218 Spanish family firm. The difference with the work of Hernández-Perlines et al. (2017) is the dependent variable: in the work of Hernández-Perlines et al. (2017) the performance of the family firm is used, while in the current study it uses international performance of the family firm: in the first case 4 items are used and in this work three types of variables are used (frequency of International activity, satisfaction of International activity, and results of internationalization). It is true that between the two types of performance the correlation is high, in this case 0.754, but it is not the same type of performance. Therefore, the second hypothesis can be stated:

**H_2_: The ACAP positively mediates the relationship between IEO and international performance of family businesses**.

The mediating model approach involves considering two relationships. On the one hand, IEO positively affects ACAP and on the other, ACAP positively influences the international performance of family businesses.

The first relationship focuses on the positive effect of IEO on ACAP (Wales et al., 2013). IEO allows generating ACAP from the identification and evaluation of new opportunities (Teng, 2007; Zahra et al., 2009; Qian and Acs, 2013). Therefore, this study can affirm that IEO becomes a background for ACAP (Brettel et al., 2011). Based on the above, the following hypothesis can be raised:

**H_2a_: IEO positively influences the ACAP of family businesses**.

Moreover, the literature review reveals plenty of studies on the positive relationship between ACAP and business profits (Mowery et al., 1996; Lewin et al., 1999; Mukherjee et al., 2000; Lane et al., 2001, 2006; Stock et al., 2001; Tsai, 2001; Zahra and George, 2002; Jansen et al., 2005; Todorova and Durisin, 2007; Bergh and Lim, 2008; Yeoh, 2009; Wales et al., 2013). Thus, the third hypothesis can be stated as follows:

**H_2b_: ACAP positively influences the international performance of family businesses**.

### Environment

The inclusion of the ENV in the model is justified by the fact that in dynamic environments companies with a high orientation toward entrepreneurship obtain better results (Wiklund and Shepherd, 2005). The EO provides additional benefits to companies when they operate in a dynamic environment (Rosenbusch et al., 2013). Miller (1983) states that the ENV and its dimensions have a positive moderating effect on the entrepreneurial orientation. Companies that adapt to dynamic environments take better advantage of opportunities presented to them (Covin and Slevin, 1989). Russell and Russell (1992) argue that dynamic and hostile environments favor the achievement of higher levels of performance. On the other hand, Wood and Robertson (1997) and Francis and Collins-Dodd (2000) affirm that the influence of the entrepreneurial orientation in the performance of the companies will be greater in dynamic and unstable environments. On the other hand, Lohrke et al. (2015)indicate that the relationship between entrepreneurial orientation and performance is moderated by market and industry factors. Also, Zahra and Garvis (2000) highlight the positive moderating effect of hostile environments on the influence of entrepreneurial orientation on corporate performance. Dimitratos et al. (2004) argue that environmental conditions have a positive moderating effect on the relationship between entrepreneurial orientation and performance. Kuivalainen et al. (2010) affirm that the competitiveness of the environment reinforces the influence of the entrepreneurial orientation on the entrepreneurial performance. For Casillas and Moreno (2010) the dynamism of the environment has a significant moderating impact on the entrepreneurial orientation. Cruz and Nordqvist (2012) the competitive ENV is strongly correlated with the entrepreneurial orientation. The competitiveness of the firm operating in turbulent environments is determined by their entrepreneurial orientation and ACAP (García-Sánchez et al., 2017). In a dynamic environment, ACAP acquires special relevance (Liao et al., 2003). The ACAP strengthens the entrepreneurial orientation relationship and the performance in turbulent markets (Engelen et al., 2014) and allows to achieve superior performance in dynamic environments (Verma et al., 2017). For Van Doorn et al. (2017) the ACAP allows to improve the understanding of the ENV when the top management of the company has a high entrepreneurial orientation.

Of the different dimensions of the ENV, in this work we have opted for hostility and dynamism for being the most studied in the literature (Covin and Slevin, 1989, 1991; Zahra, 1991; Antoncic and Hisrich, 2004).

The following moderator hypothesis exposed is a result of the previous:

**H_3_: ENV moderates the mediation of ACAP on IEO of international performance of family businesses**.

The previous moderator hypothesis may be divided into two sub-hypothesis:

**H_3a_: ENV moderates the relation between IEO and ACAP in family businesses**.
**H_3b_: ENV moderates the relation between ACAP and the international performance of family businesses**.

**Figure 1:**
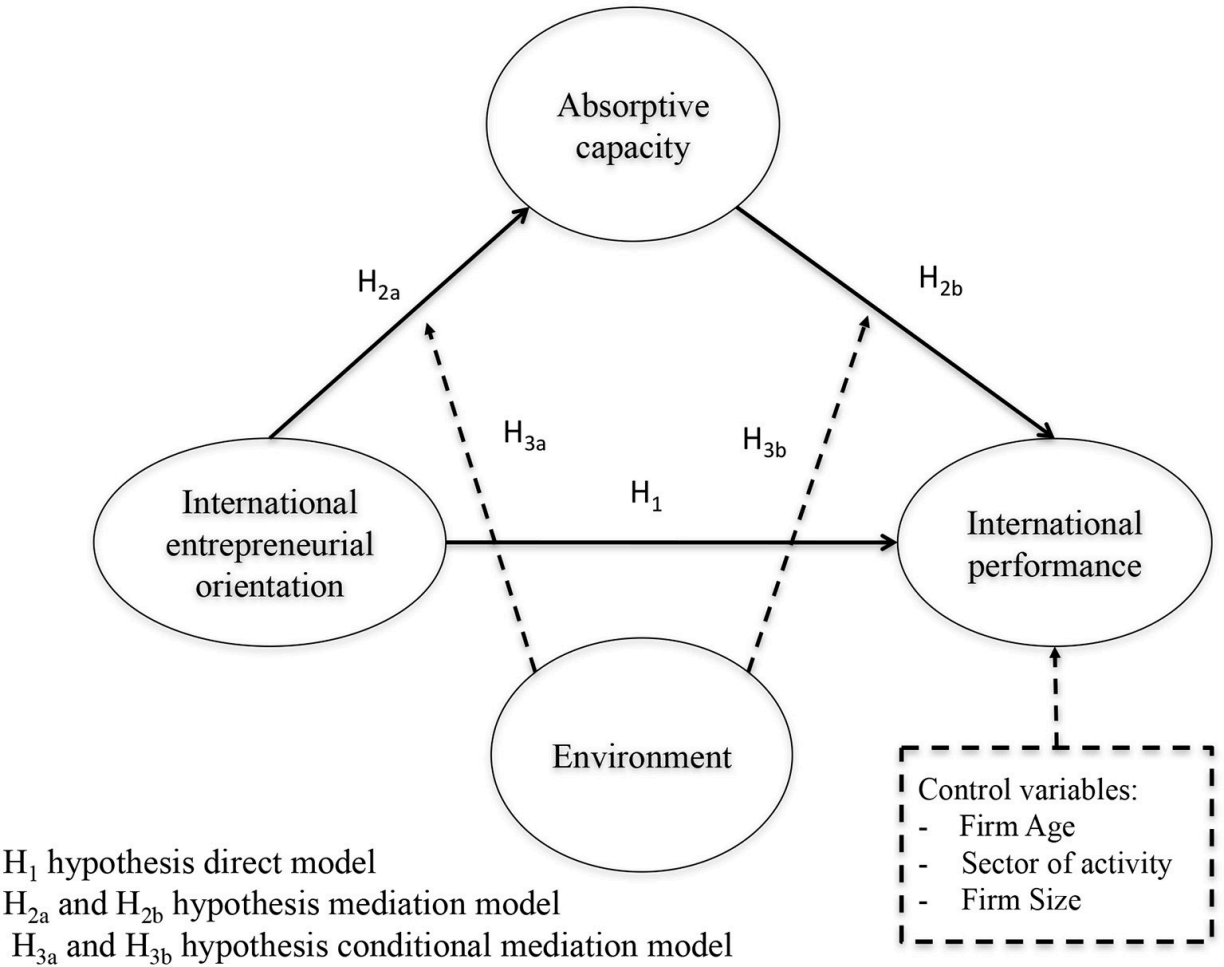
Structural model.

Once the literature review has been carried out and the corresponding hypotheses have been presented, the conceptual model is shown in Figure 1.

## Materials and methods

### Sample

Data was collected from a questionnaire sent via email -through LimeSurvey v. 2.5.- to the CEOs/Directors of a sample of companies taken from the Spanish Institute for Family Business (hereinafter IEF, as it is known in Spanish). The participation in the study was voluntary and participants were advised of the anonymity of their answers. Participants were sent a letter presenting the research and its purpose. Research Ethics Committee must not intervene in this type of studies like our research work in Spain. The questionnaire involves Likert-type questions (1–5). The sample consists of 1,045 family businesses registered in the IEF, who produced 218 responses, which represents 20.86%. Fieldwork was conducted between June to November 2016 (see Table 1).

**Table 1:** Overview of the fieldwork.

Target population (universe)	1,045 Spanish family businesses
Analysis unit/sampling unit	The company
Sample size/response rate	218 valid surveys/ 20.86%
Confidence level	95%; *z* = 1.96; *p* = *q* = 0.50; α = 0.05
Sampling error	5.91%
Key informant	CEO/director
Date of fieldwork/data collection	June–November 2016

**Figure 2:**
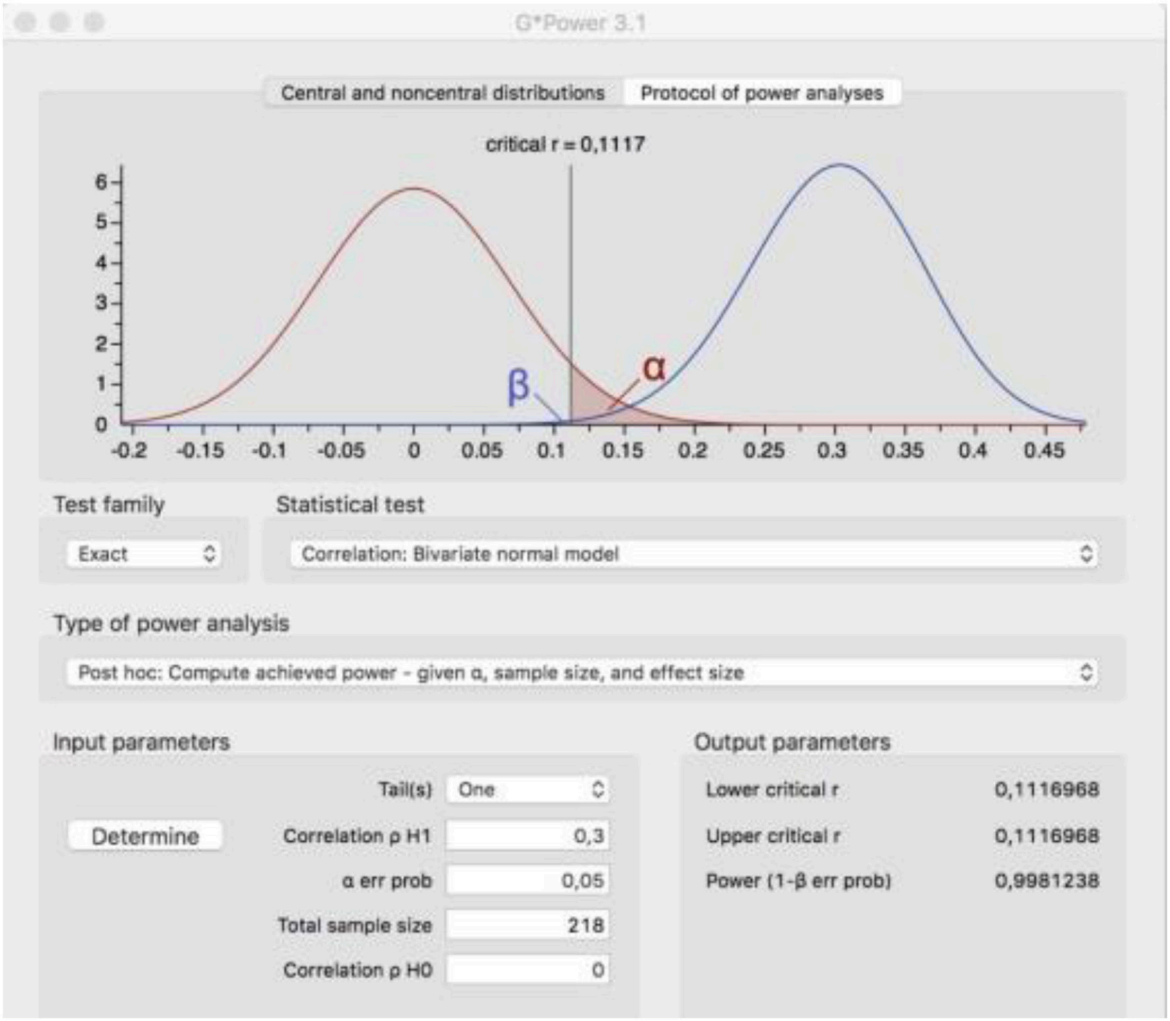
Statistical power of the sample.

The statistical power of the sample is analyzed through Cohen's retrospective test (1992). This is done using the G^*^Power 3.1.9.2 programme (Faul et al., 2009). As a result, the statistical power of the family business sample is 0.998; above the 0.80 limit established by Cohen (1992) (see Figure 2).

### Measurement of variables

#### International entrepreneurial orientation

This variable was measured according to the scale proposed by Miller (1983) and then modified by Covin and Slevin (1989), and Covin and Miller (2014). These authors think that IEO can be measured in three dimensions: innovativeness (3 items), proactiveness (3 items) and risk-taking (3 items). These variables were applied to a 5-point Likert scale.

#### Absorptive capacity

Measurement of ACAP uses a four-dimension scale validated by Flatten et al. (2011), who evaluate the extent to which the firm engages in knowledge acquisition activities (acquisition, three items), assimilates acquired information with existing knowledge (assimilation, four items), transforms recently adapted knowledge (transformation, four items), and commercially exploits knowledge transformed into competitive advantage (exploitation, three items).

#### International performance

In the present work, international performance is measured according to a multi-item scale based on exporting intensity, which was included as a measure of international performance by some authors such as Zahra et al. (1997) and Morgan et al. (2004). We also included perceived satisfaction in exporting performance, which was previously included by some authors such as Cavusgil and Zou (1994), Balabanis and Katsikea (2004), Dimitratos et al. (2004), and Zahra et al. (1997). Both previous variables were measured according to a 5-point Likert scale. Finally, the third item included to measure international performance refers to exporting results and had previously been used by some authors such as Zahra et al. (1997), Morgan et al. (2004), and Ibeh (2003).

#### Environment

Scales proposed by Robertson and Chetty (2000), Balabanis and Katsikea (2004), Dimitratos et al. (2004), Etchebarne et al. (2010), and Kuivalainen et al. (2010) have been used to measure hostility and dynamism of environment.

#### Control variables

The control variables under study are: size (number of employees), age (years of service since start-up) and activity sector (industry and services) of the family business. These variables are often used in studies on family business (Chrisman et al., 2005).

### Data analysis

In order to test hypotheses and analyze both the direct effect and the mediating effect, this study conducted the Partial Least Square (PLS) method, a structural equation multivariate quantitative method. The PLS method allows addressing the research questions, due to its predictive nature (Sarstedt et al., 2014; Hair et al., 2017); as it allows observing different causal relationships (Jöreskog and Wold, 1982; Astrachan et al., 2014) and because it is less demanding with regard to the minimum sample size (Henseler et al., 2015). The software used for data analysis through SEM-PLS was SmartPLS v.3.2.7 (Ringle et al., 2015).

## Findings

The model is analyzed and interpreted in two steps to ensure that the measurement scales are valid and reliable (Barclay et al., 1995):

1. Analysis of the measurement model;
2. Analysis of the structural model.

### Analysis of the measurement model

Following recommendations by Roldán and Sánchez-Franco (2012), the first step was to analyze composite reliability values, Cronbach's alpha and the Average Variance Extracted (AVE), in order to check the reliability of the constructs under study. The aforementioned values exceed the thresholds recommended by literature^3^, so the convergent validity of the selected scales is supported (see Table 2).

**Table 2:** Composites and indicators.

**Composite/Indicator**	**Composite reliability**	**Cronbach alpha**	**AVE**
Entrepreneurial Orientation (EO) (second-order composite mode b)	0.770	0.752	0.628
Innovativeness (first-order composite mode a)	0.864	0.766	0.682
Proactiveness (first-order composite mode a)	0.830	0.795	0.620
Risk- taking (first-order composite mode a)	0.890	0.815	0.730
Absorptive Capacity (ACAP) (second-order composite mode a)	0.837	0.837	0.664
Acquisition Capacity (first-order composite mode a)	0.921	0.871	0.796
Assimilation capacity (first-order composite mode a)	0.941	0.917	0.801
Transformation capacity (first-order composite mode a)	0.896	0.844	0.684
Exploit capacity (first-order composite mode a)	0.932	0.891	0.821
Environment (ENV) (second-order composite mode a)	0.898	0.773	0.814
Hostility (first-order composite mode a)	0.864	0.821	0.652
Dynamism (first-order composite mode a)	0.919	0.907	0.619
International performance (INTPERF) (second-order composite mode a)	0.899	0.831	0.749
Frequency of International Activity (first-order composite mode a)	0.839	0.755	0.628
Satisfaction of International Activity (first-order composite mode a)	0.906	0.870	0.659
Results of Internationalization (first-order composite mode a)	0.909	0.851	0.770

Discriminant validity was also calculated, which measures the extent to which a compound is truly different from other compounds (Hair et al., 2017). To do so, AVE square roots values for each compound were compared with correlations between constructs associated with the construct in question (Fornell and Larcker, 1981). Results show that AVE values are higher than the corresponding correlations in all cases (see Tables 3–6).

**Table 3:** Discriminant validity of entrepreneurial orientation.

	**Innovativeness**	**Proactiveness**	**Risk taking**
Innovativeness	0.825^*^		
Proactiveness	0.608	0.787^*^	
Risk-taking	0.440	0.605	0.854^*^

* *AVE square root has been calculated on the diagonal*.

**Table 4:** Discriminant validity of absorptive capacity.

	**Acquisition**	**Assimilation**	**Transformation**	**Exploit**
Acquisition	0.891^*^			
Assimilation	0.685	0.894^*^		
Transformation	0.665	0.637	0.827^*^	
Exploit	0.610	0.574	0.599	0.906^*^

* *AVE square root has been calculated on the diagonal*.

**Table 5:** Discriminant validity environment.

	**Dynamism**	**Hostility**
Dynamism	0.786^*^	
Hostility	0.607	0.807^*^

* *AVE square root has been calculated on the diagonal*.

**Table 6:** Discriminant validity of international performance.

	**Frequency of international activity**	**Satisfaction of international activity**	**Results of internationalization**
Freq. of Int. Act.			
Satisf. of Int. Act.	0.615^*^		
Results of Inter.	0.518	0.605^*^	

* *AVE square root has been calculated on the diagonal*.

In addition, the HTMT index can be calculated for type A compounds. This index measures discriminant validity among indicators of the same compound and among indicators of different compounds. For discriminant validity to occur, HTMT values must be below 0.85 (Henseler et al., 2015) (see Table 7).

**Table 7:** Heterotrait-Monotrait (HTMT) Ratio.

**Composite/Measures**	**1. IEO**	**2. ACAP**	**3. ENV**	**4. INTPERF**
1. International Entrepreneurial Orientation (IEO)				
2. Absorptive Capacity (ACAP)	0.245			
3. Environment (ENV)	0.201	0.262		
4. International Performance (INTPERF)	0.354	0.293	0.226	

Finally, HTMT_inference_ is calculated based on bootstrapping (5000 subsamples). There is discriminant validity when the resulting interval contains values lower than 1. This is the case of this study (see Table 8).

**Table 8:** HTMT_inference_.

	**Original data (O)**	**Data average (M)**	**5.0%**	**95.0%**	**Data average (M)**	**Bias**	**5.0%**	**95.0%**
International Entrepreneurial Orientation -> International Performance	0.223	0.229	0.082	0.448	0.229	0.005	0.084	0.454
International Entrepreneurial Orientation -> Absorptive Capacity	0.761	0.759	0.685	0.837	0.759	−0.002	0.683	0.835
Absorptive Capacity -> International Performance	0.092	0.075	0.043	0.326	0.075	−0.016	0.059	0.310
Environment-> International Performance	0.122	0.120	0.025	0.198	0.120	−0.002	0.012	0.224
Environment -> Absorptive Capacity	0.112	0.111	0.258	0.558	0.111	0.000	0.026	0.199

Entrepreneurial orientation became operational as a second-order type B compound, calculated in two stages based on scores of latent variables (Wright et al., 2012). In order to validate the entrepreneurial orientation compound, this study took into account recommendations by Diamantopoulos et al. (2008). In the case of a second-order type B compound, the items involved should not present any collinearity problem (Diamantopoulos and Winklhofer, 2001). Collinearity problems may only occur when the Inflation Variance Factor (IVF) reaches or exceeds the value 5 (Kleinbaum et al., 1988). In this case, no collinearity problems were observed (see Table 9).

**Table 9:** Collinearity of entrepreneurial orientation.

**Factor**	**Loads (λ)**	**FIV**
Innovation	0.384	1.602
Proactivity	0.361	1.987
Risk taking	0.424	1.563

### Analysis of the structural model

Once the convergent validity and the discriminant validity of the measurement model were confirmed, testing of the relationships between variables took place. In order to find out the effects, this study followed the steps suggested by Hair et al. (2017).

**Figure 3:**
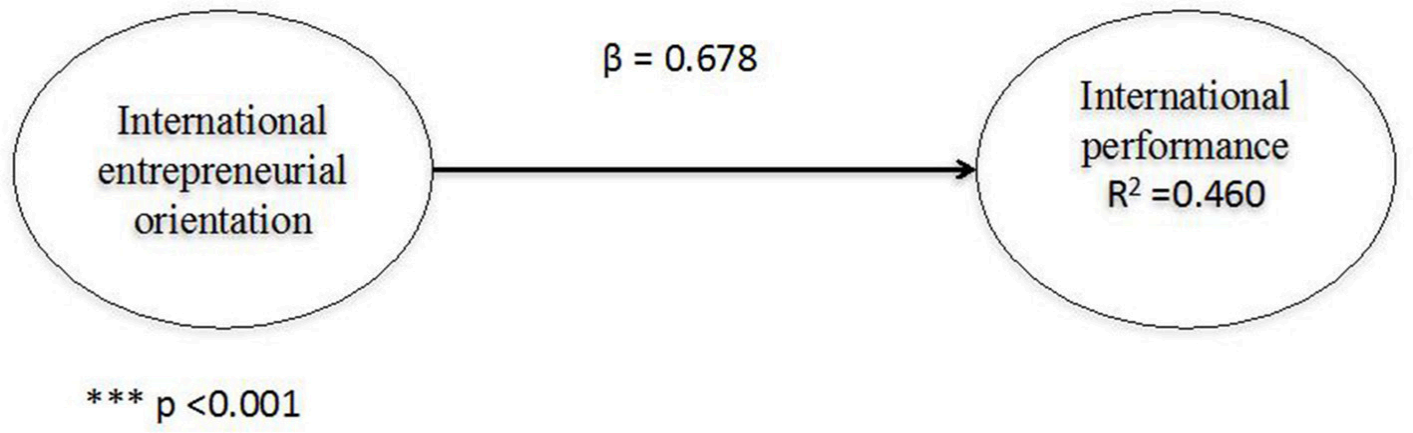
Direct model.

First, the direct effect between IEO and international performance of family businesses was analyzed. To do so, the value of the path coefficient was checked, along with its significance (by applying the bootstrapping procedure of 5,000 resamples). The effect is positive and significant (β = 0.419; *p* < 0.001) (see Figure 3 and **Table 11**). Of the model it appears that the IEO explains 32.5% of the international performance variance of family businesses.

**Figure 4:**
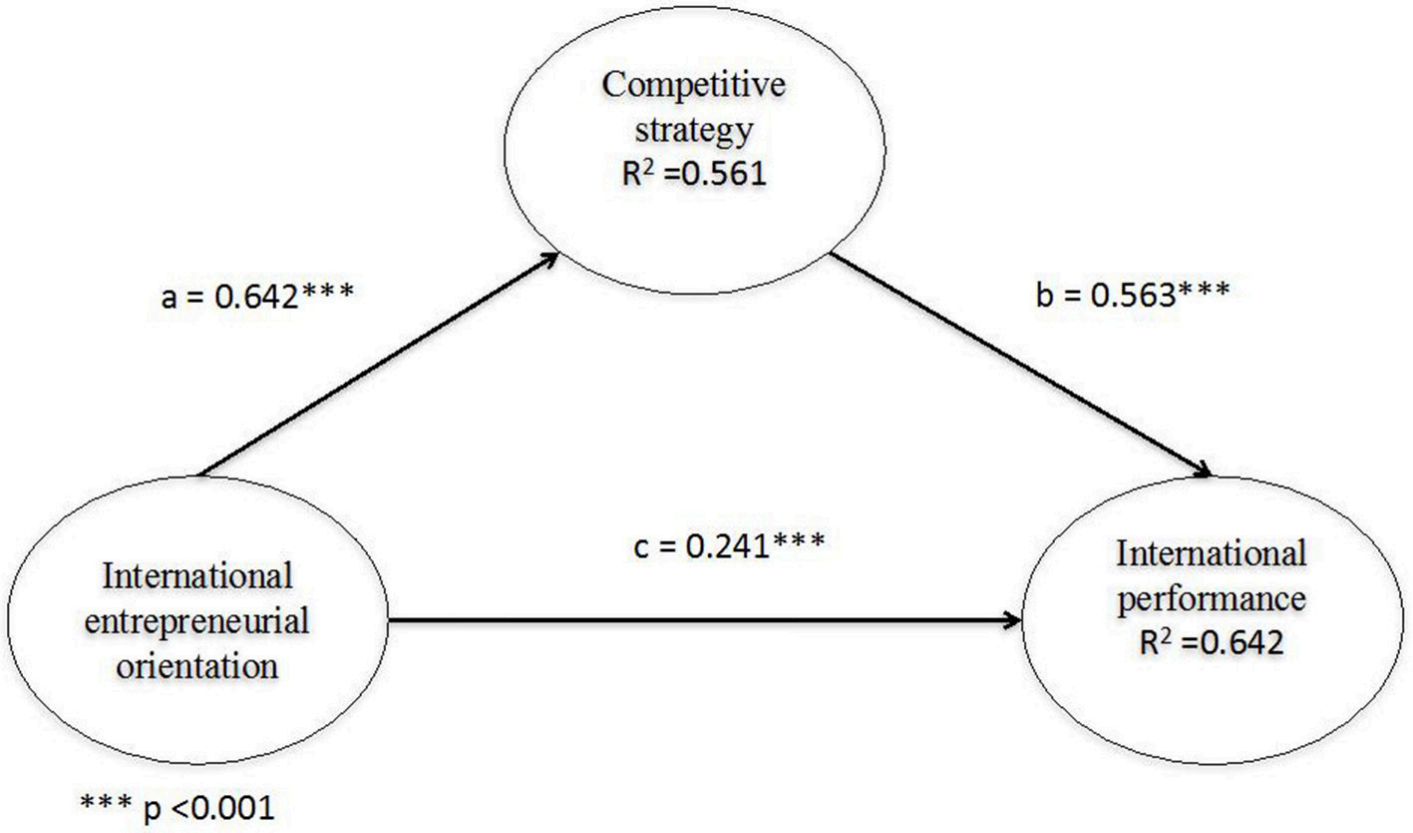
Mediation model.

The second step was to include the effect of the mediating variable (ACAP). As observed in Figure 4 and **Table 11**, the indirect effect is positive and significant (between IEO and ACAP) H_2a_: β = 0.696; *p* < 0.001; and between ACAP and international performance H_2b_: β = 0.439; *p* < 0.001). The mediating effect completely eliminates the direct effect, since the direct relationship between the IEO and the international performance of family businesses has a β = −0.093 and is not significant.

In the model, IEO explains 38.3% of the variance of ACAP, which, in turn, explains 40.6% of the variance of international performance of family businesses (see Figure 4 and Table **11**). Therefore, this study has proven the mediation between ACAP in relation to IEO and international performance of family businesses. Additionally, this mediating effect is a total effect, as it removes the direct effect (Baron and Kenny, 1986; Cepeda et al., 2016).

**Figure 5:**
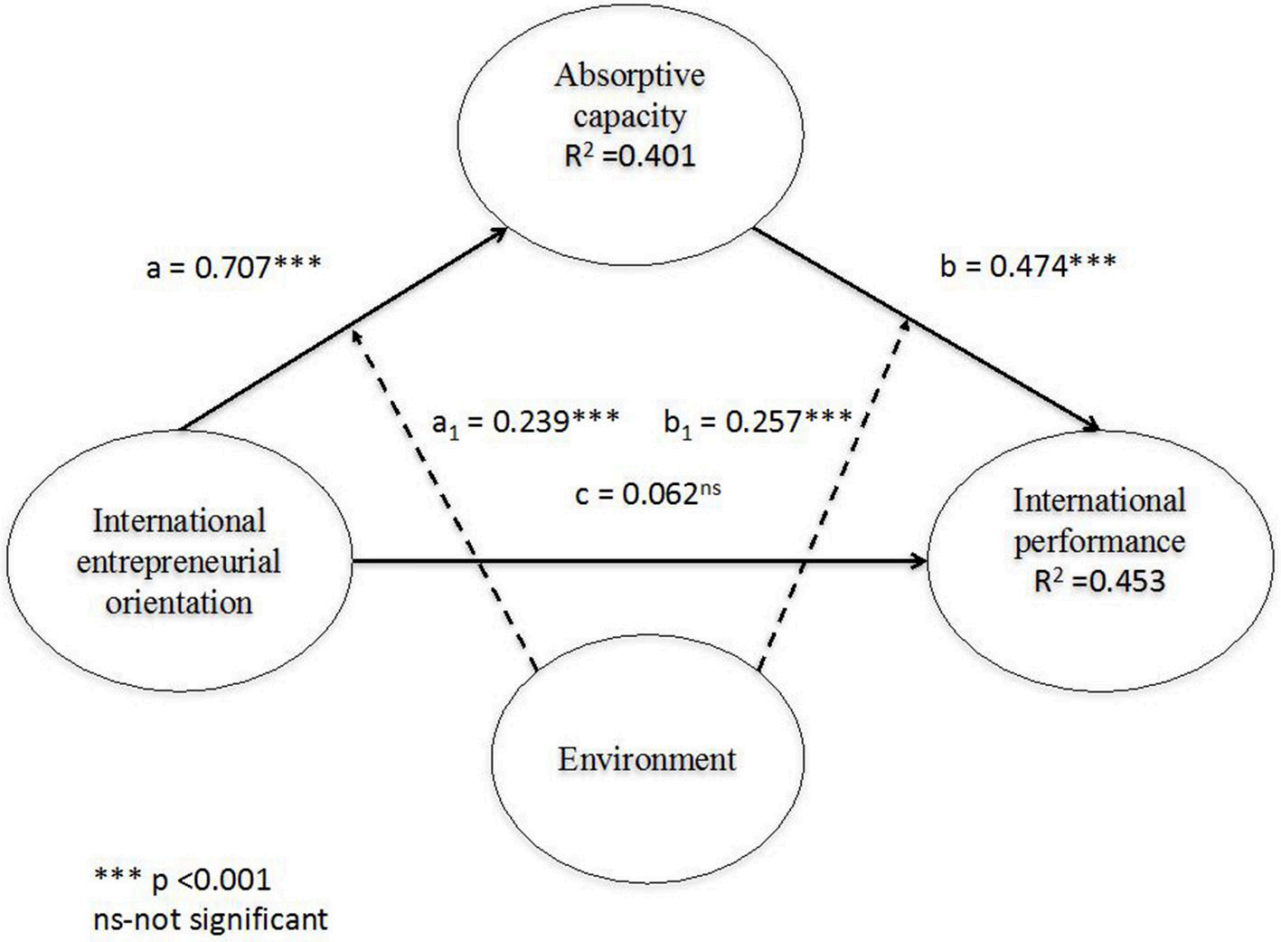
Conditional mediation model.

Third step consists on introducing moderator effect of ENV when comparing IEO vs. ACAP (first moderator effect) as well as in the relation between ACAP and international performance (second moderator effect). As we can see, moderator effect of ENV is present both in the relation IEO vs. ACAP (β = 0.239; *p* < 0.001) and ACAP vs. international performance (β = 0.257; *p* < 0.001). Moreover, this moderator effect increases explained the variance of international performance almost in 8%, from 40.6 to 45.3%, confirming the double moderator effect of ENV of the proposed model (see Figure 5). Eventually, we observe that the magnitude of moderator effect of entrepreneurial orientation is average (Chin, 2010) with a value for the first moderator effect of environment *f*^2^ = 0.19 (environment moderates the relation between IEO and ACAP) and a result of 0.24 for the second moderator effect of environment (ENV moderates the relation between ACAP and international performance).

None of the control variables has an influence that may be considered relevant (the path coefficients are less than 0.2) and are not significant (the value of *t* is lower than recommended (*p* < 0.001) (see Table 10).

**Table 10:** Control variables.

**Variable**	**ß**	***t*-value**
Firm age	0.069	0.698
Sector	−0.089	0.916
Firm size	0.115	0.571

The comparison of the two models—taking into account quality parameters—shows that the mediation model is better than the direct model: the Standardized Root Mean Square Residual (SRMR) is improved^4^. The direct model obtains a 0.073 SRMR, whereas the mediation model obtains a 0.061 SRMR, and the conditional mediation model obtains a 0.057 SRMR. Both values are below the threshold set by Henseler et al. (2015). A summary of the results and of hypotheses can be seen in Table 11.

**Table 11:** Structural model.

	**Path coefficient (β)**	***t*-value (bootstrap)**	***R*^2^**	**Support**
**Direct model (SRMR cfm** = **0.073)**				
H_1_ = IEO → INTPERF	0.419^***^	5.856	0.325	Yes
**Mediation model (SRMR cfm** = **0.061)**				
IEO → INTPERF = *c'* (direct effect of IEO on INTPERF)	−0.093^ns^	0.862		No
H_2_ = IEO → ACAP → INTPERF = *a*_1_*b*_1_ (via ACAP) (total indirect effect of EO on FPERF)	0.305			Yes
H_2a_ = IEO → ACAP = *a*_1_	0.696^***^	16.385	0.406	
H_2b_ = ACAP → INTPERF = *b*_1_	0.439^***^	8.634		
**Conditional mediation model (SRMR cfm** = **0.057)**				
IEO → INTERPERFM	c = 0.062^ns^	0.731	0.453	Yes
IEO → ACAP	a = 0.707^***^	4.048		
ACAP → INTERPERFM	b = 0.474^***^	4.362		
H_3_ = IEO*ENV → ACAP *E → INTERPERFM				
H_3a_ = IEO*ENV → ACAP	a_1_ = 0.239^***^	8.759		
H_3b_ = ACAP *ENV → INTERPERFM	b_1_ = 0.257^***^	9.823		

*** *p < 0.001; based on t(4,999), one-tailed test; ns, not significant*.

## Conclusion

The first conclusion of this model is that the dimensions used to measure the different variables and the variables themselves have values of reliability and convergent and discriminant validity above the thresholds established by the literature (Fornell and Larcker, 1981; Henseler et al., 2015; Hair et al., 2017). In addition, the models proposed have an adequate goodness of fit (the SRMR is below 0.08 recommended by Henseler et al., 2015).

The second conclusion of this study is that international performance of family businesses can be explained to a great extent by IEO. This result is consistent with previous studies (Miller and Friesen, 1983; Stetz et al., 2000; Kreiser et al., 2002; Wiklund and Shepherd, 2005; Lechner and Gudmundsson, 2014; Engelen et al., 2015; Hernández-Perlines et al., 2016a). In the direct model suggested, IEO explains 32.5% of the variance of international performance of family businesses. These results show that the first hypothesis is met, so the international presence of the firm will be determined by the firm's ability to detect new business opportunities, which can sometimes imply an increase in risk.

The third ACAP is demonstrated: it can have a direct role (e.g., Liao et al., 2003; Engelen et al., 2014), a moderating role (e.g., Engelen et al., 2014) or a mediating role (e.g., Ferreras-Méndez et al., 2015). This mediating role of the ACAP is observed in different situations (e.g., Aljanabi et al., 2014; Leal-Rodríguez et al., 2014; Hernández-Perlines et al., 2016b). In the previous work of Hernández-Perlines et al. (2017) the ACAP mediated between IEO and the performance of family businesses. In the present work, it is shown that ACAP plays a mediating role in the relationship between IEO and the international performance of family businesses. Taking into account the ACAP allows maximizing the potential of the model, which explains the variation of international performance up to 40.6%. Therefore, this study confirms the second hypothesis and helps identify the role of ACAP as a mediator.

Finally, the ENV acts as a positive moderator in the mediation model of the ACAP between the IEO and the international performance of family firm. In our case, the consideration of the ENVsupposes to improve the explanation of the variance of the international performance of the family firms up to 45.3%. This study confirms the moderating effect of the environment, and also confirms that in turbulent environments, ACAP becomes an essential part of improving EO (Todorova and Durisin, 2007). The ACAP allows to improve the understanding of the ENV (Van Doorn et al., 2017), achieving a superior performance in dynamic environments (Verma et al., 2017). In short, in turbulent environments, ACAP reinforces the influence of entrepreneurial orientation in the performance of the company (Engelen et al., 2014)

The implications of this study in relation to family business management are as follows:

1. Entrepreneurial behavior is an important factor in the international performance of family businesses (in line with Hernández-Perlines et al., 2016a).
2. IEO acts as a background for ACAP (in line with Wales et al., 2013).
3. Taking into account ACAP will allow family businesses to improve their international profits [in similar terms with what stated by Yeoh (2009)].
4. Family businesses can use the mediation of ACAP to improve their international profits through IEO, as a mediating variable.

As for limitations of the study, the first limitation is using a single informant in Likert-type scales. To overcome this limitation, the study follows recommendations by Rong and Wilkinson (2011), Woodside (2013), and Woodside et al. (2015), who suggest the appropriate selection of the person in the company to whom the questionnaire is addressed (i.e., senior executive, as recommended by Dal Zotto and Van Kranenburg, 2008). Questionnaires were sent through a computer process [e. g. email, as recommended by Torchiano et al. (2013)]. Emails requested participation, explained the research objectives and facilitated an email address to contact in case there were any questions. Furthermore, participants received emails to remind them to complete the questionnaire. The second limitation is the sample of businesses used. These are firms registered in the Spanish Institute for Family Business (IFE). The use of other databases such as SABI (Iberian Balance Sheets Analysis System) is also suggested.

As future research lines, this study suggests conducting longitudinal studies to analyze the effect of time or the presence of family members in business management, or the generational level. Also, comparative studies with other firms/countries are suggested, in order to check for significant differences. Finally, this study suggests analyzing the mediating effect of ACAP by taking into account both its potential and realized dimensions (Zahra and George, 2002).

## Ethics statement

Ethics approval for this research was not required as per institutional and national guidelines. Consent from all research participants was obtained by virtue of survey completion.

## Author contributions

All authors listed, have made substantial, direct and intellectual contribution to the work, and approved it for publication.

### Conflict of interest statement

The authors declare that the research was conducted in the absence of any commercial or financial relationships that could be construed as a potential conflict of interest.

## Footnotes

^1^This study highlights four approaches to analyze the process of business internationalization: process approach, approach of resources and capabilities, network approach and international entrepreneurship approach.

^2^The moderating effect of the environment is one of the objectives of this work.

^3^Carmines and Zeller (1979) recommend values above 0.7 for factorial loads; and Fornell and Larcker (1981) recommend values over 0.7; 0.7 and 0.5 for composite reliability, Cronbach's alpha and Average Variance Extracted values (AVE), respectively.

^4^This measure has been proposed by Hu and Bentler (1998) and Henseler et al. (2015) and allows analyzing the goodness of fit of the proposed model.
